# Description of novel capsule biosynthesis loci of *Campylobacter jejuni* clinical isolates from South and South-East Asia

**DOI:** 10.1371/journal.pone.0280583

**Published:** 2023-01-20

**Authors:** Nuanpan Khemnu, Oralak Serichantalergs, Sirigade Ruekit, Paphavee Lertsethtakarn, Frédéric Poly, Brett E. Swierczewski, John M. Crawford

**Affiliations:** 1 Department of Bacteria and Parasitic Diseases, Armed Forces Research Institute of Medical Sciences (AFRIMS), Bangkok, Thailand; 2 Enteric Diseases Department, Naval Medical Research Center, San Diego, MD, United States of America; Health Directorate, LUXEMBOURG

## Abstract

*Campylobacter jejuni* is a major cause of bacterial diarrhea worldwide and associated with numerous sequela, including Guillain-Barré Syndrome, inflammatory bowel disease, reactive arthritis, and irritable bowel syndrome. *C*. *jejuni* is unusual for an intestinal pathogen in its ability to coat its surface with a polysaccharide capsule (CPS). The genes responsible for the biosynthesis of the phase variable CPS is located in the hypervariable region of *C*. *jejuni* genome which has been used to develop multiplex PCR to classify CPS types based on the Penner serotypes. However, there still are non-typable CPS *C*. *jejuni* by the current multiplex PCR scheme. The application of the next generation sequencing and whole genome analysis software were used for the identification of novel capsule biosynthesis of *C*. *jejuni* isolates. Unique PCR primers were designed to identify these new capsule biosynthesis loci. The designed primers sets were combined in a new multiplex mix called epsilon. The unique sequences provide an additional information of the biosynthesis loci responsible for some of the common CPS sugars/residues such as heptose, deoxtyheptose and MeOPN among *C*. *jejuni* in this new group of CPS multiplex assay. This new primer complements the current *C*. *jejuni* multiplex capsule typing system and will help in identifying previously untypeable capsule locus of *C*. *jejuni* isolates.

## Introduction

*Campylobacter jejuni* represents the major *Campylobacter* species causing diarrhea in humans [1]. General bacterial strain typing systems are based on the principle that clonally related isolates share characteristics that can be tested to differentiate them from unrelated isolates using phenotypic and genotypic characterization [2]. The phenotypic methods to differentiate *Campylobacter* isolates are biotyping, serotyping, and multilocus enzyme electrophoresis. The Penner scheme is a heat-stable (HS) serotyping method developed in the early 1980s [3,4]. The serotyping scheme represents the gold standard for *Campylobacter* serotyping for over 30 years. It identifies a total of 47 *C*. *jejuni* serotypes. The scheme is based on a passive hemagglutination assay which has been used extensively in epidemiological studies for many years employing soluble antigen extracts from isolates and antisera specific to the antigens of *Campylobacter*. It was not until the early 2000s that the capsule polysaccharide was identified as the primarily heat stable bacterial structure responsible in the typing scheme [5,6]. The complexities of the assay and associated expense of producing the specific antisera have limited its usefulness as HS antigens/antibodies are not available in all laboratories. Commonly used genotypic typing methods include multilocus sequence typing (MLST) [6,7], rapid pulsed-field gel electrophoresis (PFGE) [6,8], ribo-typing [9], flagellin typing [10], amplified fragment-length polymorphism (AFLP) [11] and multiplex capsule PCR [12,13]. The multiplex PCR technique is easy to reproduce, highly discriminatory, and amenable to be performed in most laboratories.

The polysaccharide capsule (CPS) mediates the interaction between *Campylobacter* and the environment and, more importantly, contributes to its virulence [14]. The CPS biosynthesis genes are located in hypervariable regions of the *C*. *jejuni* genome [13]. The high degree of variability of CPS genes is consistent with CPS being the major serodeterminant of the Penner serotyping scheme [5]. Capsule multiplex PCR typing was developed to classify 47 Penner serotypes into 37 CPS types [12,13]. Developing a new generation of efficient tools for epidemiological surveillance of *C*. *jejuni* is clearly necessary and a high priority to overcome the limitations of available typing methods. Poly *et al*., 2013 conducted a systematic review to estimate the frequency and distribution of Penner serotypes associated with cases of Campylobacteriosis [15]. In total, more than 21,000 sporadic cases of *C*. *jejuni* cases were identified from PubMed published data between 1982 and 2011, nearly 14% of isolates were non-typable by the Penner scheme globally, a likely consequence of the fact that CPS expression in *C*. *jejuni* is known to be phase variable and successful typing in the Penner scheme requires an expression of CPS [15]. A previously published work by Poly *et al*., 2015, reported 2% from a total of 990 *C*. *jejuni* isolates collected from South and Southeast Asia from 1998–2010 were not typeable [12,13].

In this study, we aimed to characterize untypeable *C*. *jejuni* isolates from diarrhea surveillance studies conducted in South and Southeast Asia using next generation sequencing. The generated sequences were used to develop unique multiplex PCR primers and added to the existing *C*. *jejuni* CPS multiplex PCR typing assay with the goal of to expand typing coverage.

## Materials and methods

### Sources of clinical *C*. *jejuni* isolates

*Campylobacter jejuni* isolates used in this study were isolated from diarrhea etiology surveillance studies in South and Southeast Asia during 1998–2013 including military personnel participating in the 2015 Cobra Gold US-Thai joint military exercise performed in the Chonburi province, Thailand, travelers’ diarrhea, and local adult and pediatric population in Bhutan, Cambodia, Nepal, and Thailand. The isolates were untypeable by the CPS multiplex PCR assays and were selected from previous publication and from archived isolates in our laboratory [12,13]. The original studies received approval from the ethical review committees at Walter Reed Army Institute of Research (WRAIR) IRB and local IRB from their respective nations. All of the civilian participants of the stated studies provided written informed consents and US military personnel consents were obtained verbally. Parents or guardians of minors provided the consent. The isolates were de-identified archived frozen *C*. *jejuni* isolates from stool samples with appropriate consent for samples donation for future use that have been stored at the Department of Bacterial and Parasitic Diseases, AFRIMS without any identifiable information. These studies were closed. Data included in the analysis portion of this study are demographic (age and gender), clinical (associated symptoms), and laboratory (results) data which does not include any confidential or sensitive data.

### Bacterial strains, growth conditions, and genomic extractions

A total of 20 *C*. *jejuni* isolates confirmed by *lpxA* species-specific PCR [16], of unknown capsule type as they were negative by *C*. *jejuni* PCR multiplex PCR assays, were evaluated. Strains were cultured at 37°C under microaerobic conditions 5% O_2_, 10% CO_2_, and 85% N_2_ on blood agar plates. The strains *Campylobacter* spp. used to validate the multiplex PCR including *C*. *coli* (NCTC 11353), *C*. *concisus* (ATCC33237), *C*. *curvus* (ATCC35224), *C*. *fetus* (ATCC27374), *C*. *gracilis* (ATCC33236), *C*. *helvaticus* (ATCC 51209) *C*. *hyointestinalis* (ATCC35217), *C*. *jejuni* (ATCC81176), *C*. *lari* (ATCC35221), *C*. *mucosalis* (ATCC43264), *C*. *rectus* (ATCC33238), *C*. *showae* (ATCC51146), *C*. *sputorum* (ATCC35980),*C*. *upsaliensis* (ATCC43954), and *C*. *ureolyticus* (ATCC43954) were grown following ATCC instructions. Genomic DNA were extracted using the Wizard^®^ Genomic DNA Purification Kit (Promega, Madison, WI, USA) according to the manufacturer’s instructions. Genomic DNAs were stored at − 70°C until processed.

### Genome sequencing and CPS locus identification

Genomic DNA of all *C*. *jejuni* isolates were sequenced at Tufts University Core Facility (TUCF) Genomics at Tufts University Boston, MA, USA. The quality and quantity of input DNA samples were validated with Agilent Fragment Analyzer and by ThermoFisher Qubit 3 Fluorometer with Qubit dsDNA HS Assay kit. The DNA samples were sheared using a Covaris M220 focused-ultrasonicator. DNA sequencing libraries were prepared from each sample with Illumina PCR-free DNA Sample Preparation kit with 1 μg of sheared DNA as input. The prepared libraries were pooled and quantified with a MiSeq Nano 300-cycle kit, followed by a full-scale sequencing with a MiSeq V2 500 cycles kit with paired-end 250 format. The raw sequencing data were processed into demultiplexed fastq files with Illumina bcl2fastq software. The Illumina FASTQ paired-read data were transferred using parameter of quality score as NCBI/Sanger or Illumina Pipeline 1.8 and later then assembled into contig using De Novo Assembly 1.3 with a default setting on CLC Genomics workbench software version 9.5.3 (Qiagen, Denmark, https://resources.qiagenbioinformatics.com/manuals/clcgenomicsworkbench/current/index.php?manual=De_Novo_Assembly.html). The de novo assembly algorithm in QIAGEN CLC Genomics Workbench utilizes de Brujin graphs to represent overlapping reads which is a common approach for short read de novo assembly that allows a large number of reads to be handled efficiently [17–19]. Parameters used: Word size: 21, Bubble size: 251, Minimum contig length: 1,000. Assembled contigs were mapped back to contigs (slow) using Mismatch cost: 2, insertion cost: 3, Deletion cost: 3, length fraction: 50%, and Similarity fraction: 95%. The assembled genomes with contig longer than 1 kb were submitted to GenBank. All genome sequences were submitted to the PubMLST *C*. *jejuni*/ *C*. *coli* databases https://pubmlst.org/organisms/campylobacter for MLST typing and novel STs were submitted to this database. Genome sequences of all isolates were subjected to a simple BLAST to locate *kpsC* and *kpsF*, the genes harboring *C*. *jejuni* biosynthesis variable locus (http://blast.ncbi.nlm.nih.gov/Blast.cgi) [20]. Open reading frame (ORF) of the capsule locus of each isolate were generated by Rapid Annotation using Subsystem Technology (RAST) server (http://rast.nmpdr.org) with annotation scheme: ClassicRAST and default setting. A database was created containing CPS gene clusters of all of the *C*. *jejuni* isolates in this study. The Artemis comparison tool (ACT) software [21] was used to compare the CPS locus between the most similar Penner serotype and CPS sequences of representative untypeable *C*. *jejuni*.

### PCR primer design

Unique CPS sequences of the variable capsule biosynthesis region located between *kpsC* and *kpsF* were identified by BLAST on NCBI website (http://blast.ncbi.nlm.nih.gov/Blast.cgi) against all downloaded reference Penner serotypes of *C*. *jejuni* capsule loci from GenBank (http://www.ncbi.nlm.nih.gov/). The sequences were managed on MEGA version 7 [22] to define the unique regions location of each isolated gene for multiplex primer design. Multiplex primers were designed via the online software Primer 3 [6] with the following parameters: length between 18 and 30 residues, 20 to 50% GC, Tm ranging from 57 to 63°C. The sequences and gene location of primers were designed and are displayed in Table 1. All primers were compared to *C*. *jejuni* genomes via NCBI BLAST software to exclude potential amplification outside of the CPS locus. CLC genomics workbench was used to predict the size of PCR product of each primer pair on variable capsule region of untyped CPS sequences. The primers were evaluated by PCR detection of these unknown capsule types, CPS types included in the alpha, beta, gamma and delta CPS mixes of the multiplex PCR assay that identified all of the 47 Penner serotypes. ATCC *Campylobacter* spp. were also included.

**Table 1:** Information of capsule multiplex-PCR primers.

Group	ORF/ Sequence 5’ to 3’	Expected size
1	Glycosyltransferase	999
	**Forward** ACGGCAATAATCCCACCAAA	
	**Reverse** TGGGCGCTTGAGCATTTGA	
2	CMP-N-acetylneuraminate-beta-galactosamide- alpha-2,3-sialyltransferase	768
	**Forward** ACAATGCTGATTGTTGTAGAGC	
	**Reverse** ACCCATACGCTTGGTTCTGT	
3	Capsular polysaccharide biosynthesis heptosyltransferase HddD	578
	**Forward** TGTATGCGCTGTTGATGGGA	
	**Reverse** CCAACTGAGAAAAACCCGAGC	
4	Glycosyltransferase	486
	**Forward** AAGCGAGAAATTGCATGGGT	
	**Reverse** TCACCAAAACCATCCGCTCT	
5	4-hydroxy-2-ketovalerate aldolase, putative and hypothetical protein	383
	**Forward** CTTGAGCGCAAGCCAAAGAC	
	**Reverse** TTGGTCATGATCGATGCGGT	
6	Mlr0581 protein	311
	**Forward** CGAGGGTAATTACGTTGCAGA	
	**Reverse** TGCCCCACATATACGACAAAGT	
7	Hypothetical protein	266
	**Forward** TACAATGTGGTGAATGCGGT	
	**Reverse** TGATGCAGATGGATCAACGCT	
8	Amidophosphoribosyltransferase	241
	**Forward** TTGGAGATGGGATGGCAAGA	
	**Reverse** ATTCCAATGTTACCCGCGCT	

### Multiplex PCR parameter

PCR was performed in 12.5 μL reactions containing 0.5 μM of each primer (8 pairs), 1–10 ng of DNA template, 1xPCR Gold Buffer, 2.5 mM MgCl_2_, 1.5 mM mixed dNTP and 0.65 U of AmpliTaq Gold DNA Polymerase (Thermo Fisher Scientific Baltics, UAB, Vilnius, Lithuania) using Mastercycler nexus G2 Thermocycler (Eppendorf, USA). DNA amplification was performed using an initial denaturation step at 94°C for 5 min; followed by 25 cycles of amplification (denaturation at 94°C for 1 min, annealing at 55°C for 1 min, and extension at 72°C for 1 min) and ending with a final extension at 72°C for 10 min. PCR amplicons were, then, visualized by gel electrophoresis on 2.5% agarose 1000 in 0.5× TBE (Tris-borate-EDTA) buffer at 175 V for 75 min. The unique amplicons for each untypeable *C*. *jejuni* group were determined by their size by comparison with a DNA marker, 50 bp DNA Ladder (GeneRuler, Thermo Scientific).

## Results and discussions

### Diversity of the CPS loci gene

**Fig 1:**
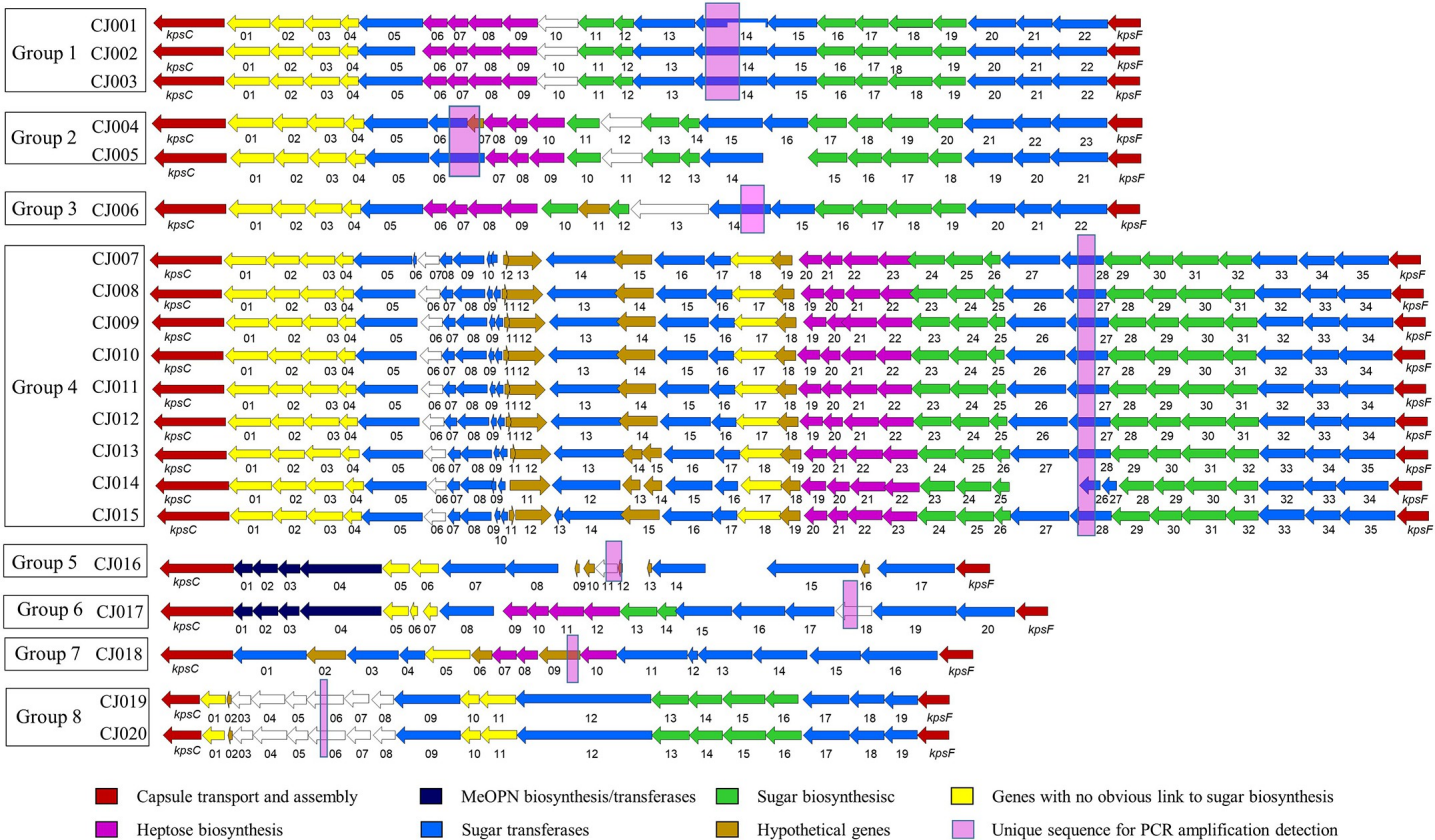
Schematic diagram of variable CPS loci of untypeable *Campylobacter jejuni* (CJ001 to CJ020) sequences. Genes are color coded as shown in the figure on the basis of best homology to any predicted proteins in databases by http://rast.nmpdr.org.

**Fig 2:**
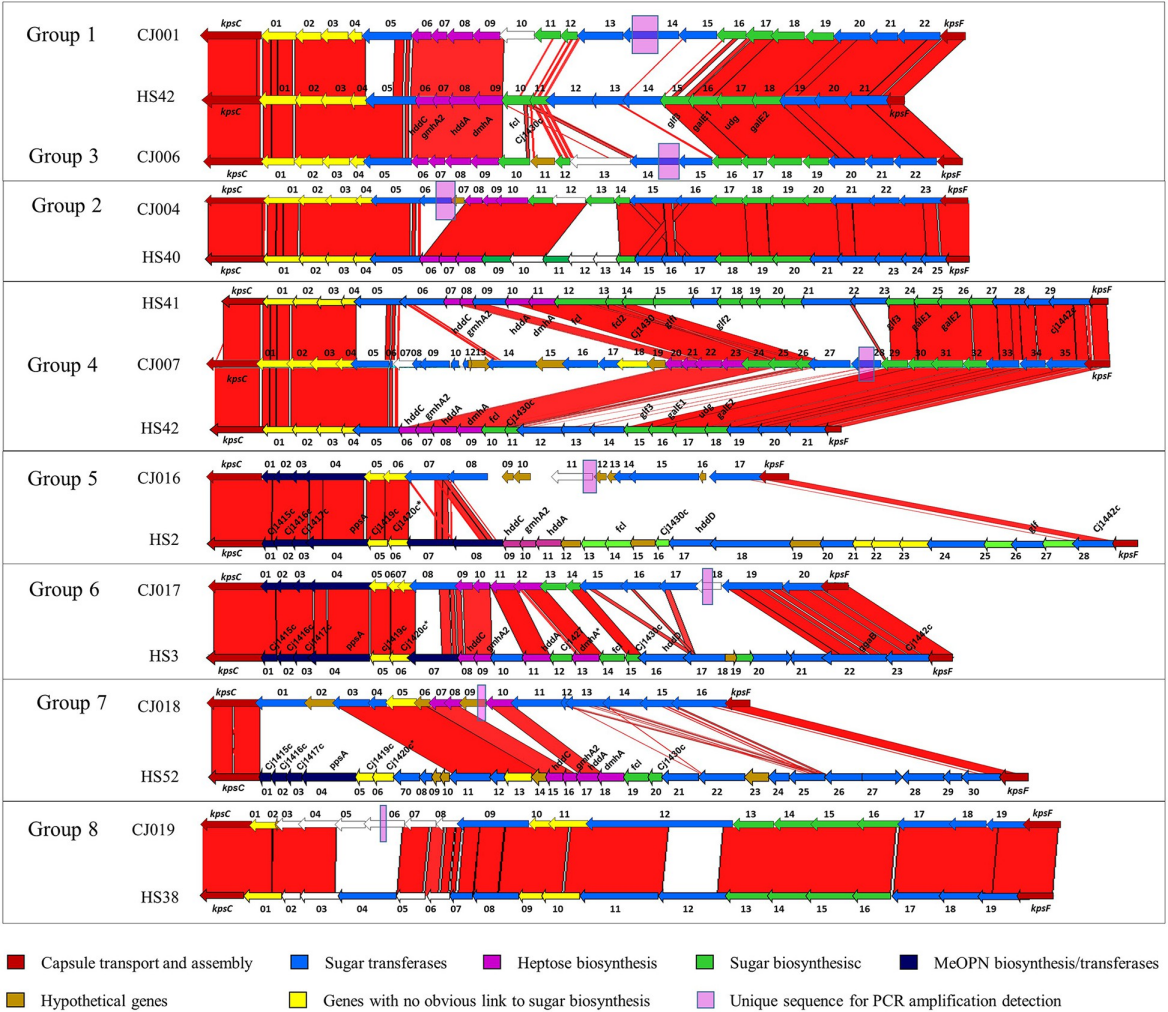
Comparison of 8 CPS sequences, representative of untypeable *Campylobacter jejuni* groups, to the most similar Penner serotype strains (Poly, F *et al*., 2015) [12]. The red vertical bars represent identical regions between loci. The outermost genes (5’ and 3’ of sequences) in red represent *kpsC* (left) and *kpsF* (right), two conserved genes in CPS synthesis that bracket the hypervariable CPS loci. The comparison was generated by the Artemis comparison tool (ACT).

All of the 20 *C*. *jejuni* genomic sequences were assembled into contigs with CLC workbench. The assembled genomes were deposited in GenBank database under Bioproject ID PRJNA690676, biosample accession number SAMN18394809 to SAMN18394828. All contigs of whole genome sequence data were subjected to BLAST for an alignment with known capsule biosynthesis genes, *kpsC* and *kpsF*. The overall data are summarized in Table 2 and Fig 1. The length of capsule loci ranged from 23,383 to 37,360 bp with 16–35 ORFs as identified by the RAST program. BLAST alignment with the capsule sequences from all 47 Penner serotypes indicated that the 20 untyped CPS were assembled into 8 groups based on their genetic similarity. Group 1 to 8 were genetically related to 7 serotypes: HS2, HS3, HS38, HS40, HS41, HS42, and HS52, respectively (Fig 2).

**Table 2:** Summary of CPS loci from sequenced *C*. *jejuni*.

Biosample_accession no.	CPS locus assigned name	Country	Size (bp)	CG (%)	No. of genes	No. of MeOPN transferase	Genes for synthesis Heptose	Deoxyheptose	No. of sugar transferases	Similar Penner serotype			MLST	Group no.
										Serotype	Query cover	Identity		
SAMN18394809	CJ001	Nepal	28624	27.1	22	0	Yes	Yes	7	HS42	66%	94%	2993	1
SAMN18394810	CJ002	Nepal	28623	27.1	22	0	Yes	Yes	7	HS42	66%	94%	2993	
SAMN18394811	CJ003	Nepal	28624	27.1	22	0	Yes	Yes	7	HS42	66%	94%	2993	
SAMN18394812	CJ004	Cambodia	27885	27.3	23	0	Yes	No	7	HS40	79%	98%	3630	2
SAMN18394813	CJ005	Nepal	27866	27.0	21	0	Yes	No	6	HS40	78%	98%	3630	
SAMN18394814	CJ006	Nepal	28509	27.5	22	0	Yes	Yes	6	HS42	70%	96%	45	3
SAMN18394815	CJ007	Cambodia	37357	27.0	35	0	Yes	Yes	14	HS41	58%	96%	986	4
SAMN18394816	CJ008	Cambodia	37356	27.0	34	0	Yes	Yes	13	HS41	58%	96%	986	
SAMN18394817	CJ009	Nepal	37358	27.0	34	0	Yes	Yes	13	HS41	58%	96%	3632	
SAMN18394818	CJ010	Nepal	37356	27.0	34	0	Yes	Yes	13	HS41	58%	96%	3632	
SAMN18394819	CJ011	Nepal	37356	27.0	34	0	Yes	Yes	13	HS41	58%	96%	986	
SAMN18394820	CJ012	Nepal	37355	27.0	34	0	Yes	Yes	13	HS41	58%	96%	986	
SAMN18394821	CJ013	Nepal	37356	27.0	35	0	Yes	Yes	13	HS41	58%	96%	3632	
SAMN18394822	CJ014	Nepal	37108	27.0	34	0	Yes	Yes	13	HS41	58%	96%	3632	
SAMN18394823	CJ015	Nepal	37360	27.0	35	0	Yes	Yes	14	HS41	58%	96%	986	
SAMN18394824	CJ016	Nepal	23383	25.9	17	2	No	No	5	HS2	44%	97%	2360	5
SAMN18394825	CJ017	Bhutan	25237	27.6	20	1	Yes	Yes	6	HS3	73%	96%	11089	6
SAMN18394826	CJ018	Bhutan	22855	25.5	16	0	Yes	No	9	HS52	34%	95%	3578	7
SAMN18394827	CJ019	Thailand	23390	27.8	19	0	No	No	5	HS38	76%	98%	11092	8
SAMN18394828	CJ020	Bhutan	23388	27.8	19	0	No	No	5	HS38	76%	98%	11093	

#### Group 1: CJ001, CJ002 and CJ003

These 3 clinical isolates were recovered from local Nepalese adults in 2008. All of the strains belong to ST 2993. The CPS biosynthesis locus size is 28.6 kb and contains 22 ORFs. The CPS loci is most similar to HS42 at 94% identity with 66% coverage (Fig 2) containing genes involved in heptose and deoxyheptose synthesis but lacking MeOPN biosynthesis and transferase genes. There are 2 regions of unique sequences identified with an approximate length of 1.1 on heptosyltransferase HddD and 5.3 kb on other heptosyltransferase HddD and Glycosyltransferase gene.

#### Group 2: CJ004 and CJ005

Both isolates were collected from symptomatic local children under 5 years old: CJ004 was collected from Cambodia in 2004 and CJ005 from Nepal in 2008. Both belong to ST 3630. The CPS biosynthesis locus is 27.8 kb and contains 23 and 21 ORFs respectively. The group 2 CPS biosynthesis locus is 98% identical to HS40 CPS locus and has the longest queried length coverage at 79%. Their sequences contain genes for heptose synthesis but lack of MeOPN biosynthesis gene, transferase and the *dmhA* gene involved in deoxyheptose biosynthesis, compared to HS40. The unique region identified is 1 kb in length (Fig 2) on CMP-N-acetylneuraminate-beta-galactosamide-alpha-2,3-sialyltransferase (EC 2.4.99.-) protein encoded gene.

#### Group 3: CJ006

CJ006 was isolated from a Nepalese child in 2007. The CPS loci is 28.5 kb long and is the most similar to HS42 like group 1. It contains genes involved in heptose and deoxyheptose synthesis, lacks MeOPN and is of ST 45. CJ006 CPS’ unique region is nearly 2.7 kb long covering Capsular polysaccharide biosynthesis heptosyltransferase HddD and Glycosyltransferase (EC 2.4.1.-) protein encoding gene.

#### Group 4: CJ007, CJ008, CJ009, CJ010, CJ011, CJ012, CJ013, CJ014 and CJ015

CJ007 and CJ008 were isolated from Cambodian children in 2004 and 2006, respectively, whereas CJ009 and CJ010 were from Nepalese children in 2008. CJ011 to CJ015 were isolated from local adults and travelers in Nepal from 2001 to 2012. The CPS loci size is 37 kb long with 34–35 ORFs. All of them are most similar to HS41 at 96% identity with 58% coverage followed by HS42 (95% identity with 56% coverage). The loci contains both genes that are involved in heptose and deoxyheptose synthesis but lack MeOPN genes. They are distinguished into 2 subgroups by MLST typing: ST 986 and ST 3632. There are 2 unique regions identified at 2.5 kb on Putative sugar transferase and 2.9 kb covering CMP-N-acetylneuraminate-beta-galactosamide-alpha-2,3-sialyltransferase (EC 2.4.99.-) and hypothetical protein encoding gene, respectively.

#### Group 5: CJ016

The isolate was isolated from a travelers’ diarrhea case in Nepal in 2001. The CPS loci is 23.4 kb long with identified 17 (ORFs). The sequence has high identity (97%) with a short coverage of 44% to HS2 CPS with a similar characteristic of containing MeOPN transferase but no heptose and deoxyheptose synthesis genes. Three unique regions were identified with an approximate length of 0.7 kb that partially covers Beta-1,3-glucosyltransferase and hypothetical protein, 3.9 kb that partially covers Beta-1,3-glucosyltransferase and CMP-N-acetylneuraminate-beta-galactosamide-alpha-2,3-sialyltransferase (EC 2.4.99.-), and 4.9 kb that covers 4-hydroxy-2-ketovalerate aldolase, putative and hypothetical protein encoding gene.

#### Group 6: CJ017

The isolate was isolated from a local diarrhea patient in Bhutan in 2013. The CPS loci is 27.6 bp long with 20 ORFs and has 96% identity with 73% sequence coverage to HS3. The CPS loci contains genes that are involved in MeOPN transferase, heptose and deoxyheptose synthesis. The novel MLST, ST11089, was identified and submitted to the PubMLST *C*. *jejuni/ C*. *coli* databases https://pubmlst.org/organisms/campylobacter. There are 3 unique regions at lengths of 0.6 that partially covers Beta-1,3-galactosyltransferase / Beta-1,4-galactosyltransferase and Capsular polysaccharide biosynthesis heptosyltransferase HddD, 1.0 kb on Mlr0581 protein, and 1.1 kb on Capsular polysaccharide biosynthesis heptosyltransferase HddD encoding gene.

#### Group 7: CJ018

The isolate was from a diarrhea case of a local Bhutanese child in 2013. The CPS loci is 22.8 kb long with 16 ORFs. It is most similar to HS58 with 95% identity but with only 34% sequence coverage. It contains deoxyheptose synthesis gene but lacks MeOPN and heptose synthesis genes. Three unique regions at 1.3, 2.4 and 3.2 kb in lengths were identified. Three unique regions at 1.3 kb covering the whole Phosphoheptose isomerase 2 (EC 5.3.1.-) and hypothetical protein, 2.4 kb that partially covers Capsular polysaccharide biosynthesis heptosyltransferase HddD and Beta-1,3-galactosyltransferase / Beta-1,4-galactosyltransferase, and 3.2 kb covering parts of CMP-N-acetylneuraminate-beta-galactosamide-alpha-2,3-sialyltransferase (EC 2.4.99.-), CMP-N-acetylneuraminate-beta-galactosamide-alpha-2,3-sialyltransferase (EC 2.4.99.-) and the whole hypothetical protein encoding gene.

#### Group 8: CJ019 and CJ020

CJ019 was isolated from US military personnel in Thailand in 2015 and CJ020 was isolated from a pediatric diarrhea patient in Bhutan in 2013. Both CPS loci are 23.4 kb long, coding for 19 ORFs, and lack all genes involved in MeOPN heptose and deoxyheptose synthesis. They are most similar to HS38 Penner serotype with 95% identity on 76% coverage. Both are of novel MLST, ST11092 and ST11093, for CJ019 and CJ020, respectively. Sequences were submitted to the PubMLST *C*. *jejuni/ C*. *coli* databases https://pubmlst.org/organisms/campylobacter. Two unique regions were identified with 1.5 kb on Putative sugar transferase and 1.8 kb in lengths on Amidophosphoribosyltransferase (EC 2.4.2.14) encoding gene.

### Design of primer and multiplex PCR development

**Fig 3:**
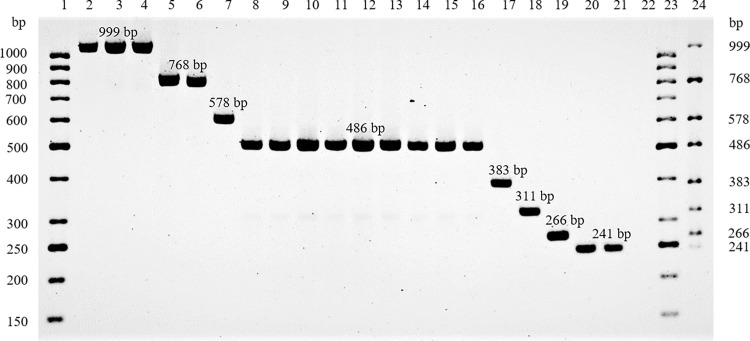
A multiplex PCR assay for 20 untypeable *C*. *jejuni*, CJ001 to CJ020. Lanes 1 and 23 are 50 bp DNA Ladder (GeneRuler, Thermo Scientific). Lanes 2 to 21 represent PCR products of CJ001-CJ020 using the 8 newly designed primer pairs based on 8 different unique sequences from each group. Lane 22 is a negative control. Lane 24 represents a mixed PCR products, a multiplex PCR assay using representative DNA template from each group.

Eight pairs of primer (Table 1) were designed using unique sequences identified. The location and PCR product size of the target genes on the unique regions were defined by the CLC workbench. The targets from unique sequences and expected amplicon size are as follows: glycosyltransferase coding gene of 999 bp for group 1, CMP-N-acetylneuraminate-beta-galactosamide-alpha-2,3-sialyltransferase coding gene of 768 bp for group 2, capsular polysaccharide biosynthesis heptosyltransferase *HddD* gene of 578 bp for group 3, glycosyltransferase coding gene of 486 bp for group 4, a region across 4-hydroxy-2-ketovalerate aldolase and a putative and hypothetical protein coding gene of 383 bp for group 5, Mlr0581 protein coding gene of 311 bp for group 6, a hypothetical protein coding gene of 266 bp for group 7, and amidophosphoribosyltransferase of 241 bp for group 8. The specificity of the amplification of these unique regions is shown in Fig 3. All 20 untypable *C*. *jejuni* produced PCR products that matched the predicted size by CLC workbench in all eight groups (Fig 3). No cross reactions were observed when these primer pairs were used in PCR with DNA extracts of positive control capsule type for alpha, beta, gamma and delta CPS by multiplex PCR assays [12] that identified all of the 47 Penner serotypes (S2A, S2B and S2C Fig). Eight pairs of primer were also tested on other ATCC *Campylobacter spp*. and no PCR products were generated indicating the specificity of the designed primers to identify these unique untypable *C jejuni* isolates (S2D Fig). This new multiplex primer mix was named epsilon.

## Conclusions

This study provides an improvement in capsule typing among untypeable *C*. *jejuni* isolates that are not described by the Penner serotype system or previously recognized by the multiplex typing system [12]. The application of next generation sequencing and utilization of sequencing data to develop multiplex PCR are useful tools in updating the molecular based CPS typing system. The herein established epsilon PCR mix applied to previously untypeable *C*. *jejuni* clinical as well as environmental isolates will provide additional information on circulating *C*. *jejuni* CPS types.

While the sequencing and PCR primers do not establish the CPS structures, it nevertheless provides information on the potential biosynthesis of common *C*. *jejuni* CPS sugars/residues including biosynthesis of heptose, deoxyheptose and MeOPN.

## Supporting information

S1 Fig The original image of Fig 3. A mutiplex PCR assay for 20 untypeable C. jejuni, CJ001 to CJ020.
(PDF)

S2 Fig A multiplex PCR assay using epsilon primers on *Campylobacter* spp. DNA of capsule types alpha, beta, gamma and delta CPS that were identified to match all of the 47 Penner serotypes and other ATCC *Campylobacter spp*.
(PDF)

S1 Table Capsule biosynthesis loci of 20 *Campylobacter jejuni* clinical isolates from South and South-East Asia and closet relationship to known Penner type. (XLSX)
