# Supplementary material for: Description of novel capsule biosynthesis loci of Campylobacter jejuni clinical isolates from South and South-East Asia
Source: PLoS One. 2023 Jan 20;18(1):e0280583. doi: 10.1371/journal.pone.0280583 (PMC9858101; doi:10.1371/journal.pone.0280583)
Supplement: S1 Fig — A mutiplex PCR assay for 20 untypeable C. jejuni, CJ001 to CJ020. (PDF) [file pone.0280583.s001.pdf]

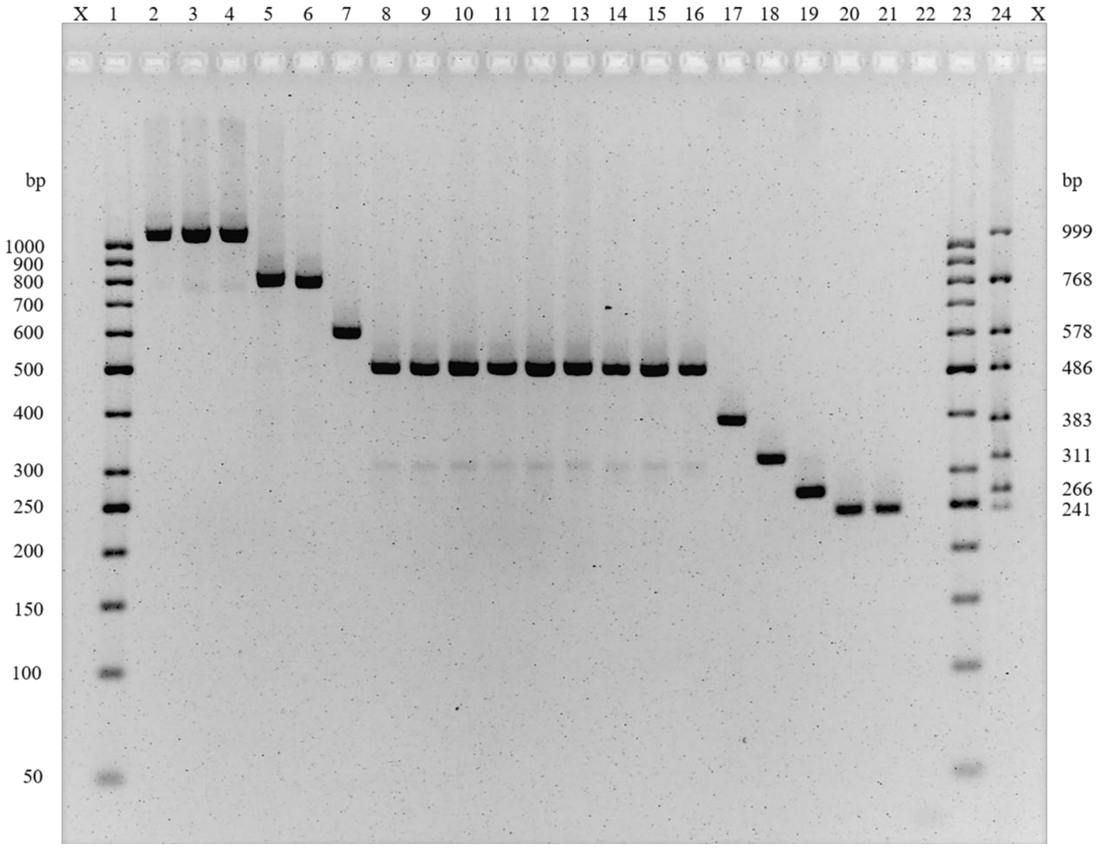

The original image of Fig 3. A Multiplex PCR assay for 20 untypeable *C. jejuni*, CJ001 to CJ020. Lanes 1 and 23 are 50 bp DNA Ladder (GeneRuler, Thermo Scientific). Lanes 2 to 21 represent PCR products of CJ001-CJ020 using the 8 newly designed primer pairs based on 8 different unique sequences from each group. Lane 22 is a negative control. Lane 24 represents a mixed PCR products, a multiplex PCR assay using representative DNA template from each group.
