# Supplementary material for: Description of novel capsule biosynthesis loci of Campylobacter jejuni clinical isolates from South and South-East Asia
Source: PLoS One. 2023 Jan 20;18(1):e0280583. doi: 10.1371/journal.pone.0280583 (PMC9858101; doi:10.1371/journal.pone.0280583)
Supplement: S2 Fig — DNA of capsule types alpha, beta, gamma and delta CPS that were identified to match all of the 47 Penner serotypes and other ATCC Campylobacter spp. (PDF) [file pone.0280583.s002.pdf]

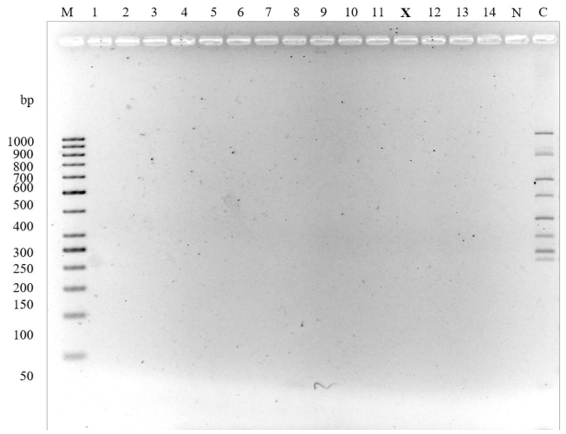

(A)

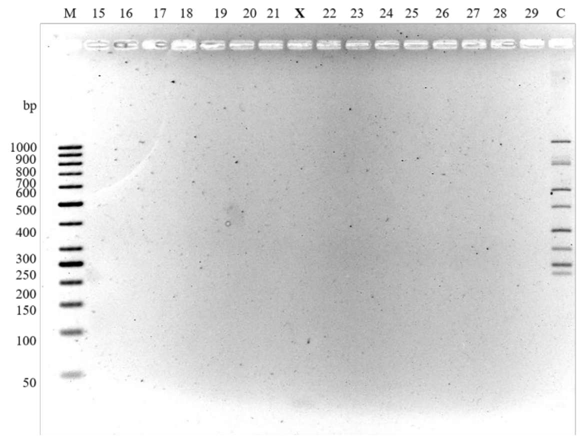

(B)

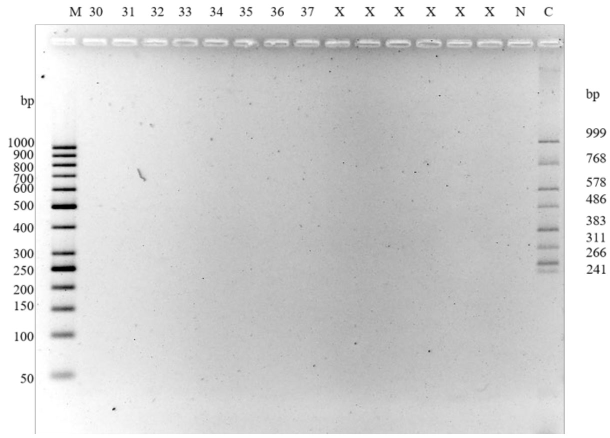

(C)

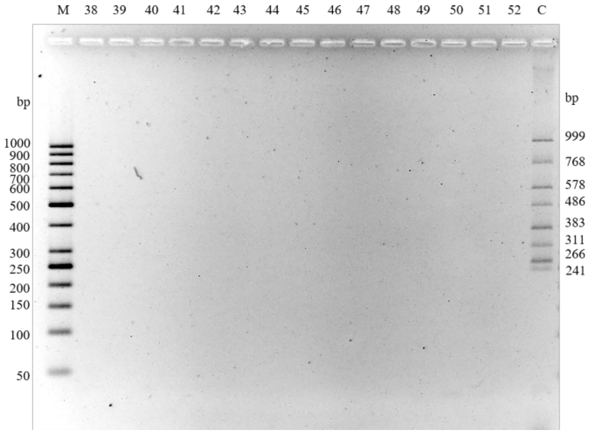

(D)

S2 Fig. A multiplex PCR assay using epsilon primers on Campylobacter spp. DNA of capsule types alpha, beta, gamma and delta CPS that were identified to match all of the 47 Penner serotypes and other ATCC Campylobacter spp. (A), (B) and (C); Lanes 1-11 are of the alpha mix: HS2, HS3, HS4, HS6, HS10, HS15, HS41, HS53, HS19, HS63 and HS33 respectively. Lanes 12-21 are of the beta mix: HS4, HS23, HS17 and 8, HS1 HS42, HS21, HS31, HS12, HS27 and HS57 respectively. Lanes 22-29 are of the delta mix: HS60, HS32, HS58, HS11, HS40, HS52, HS55 and HS38 respectively. Lanes 30-37 are of the gamma mix HS37, HS22, HS44, HS9, HS18, HS29, HS45 and HS62 respectively. (D); Other ATCC *Campylobacter spp*. in lanes 38-52 in following order: *C. concisus*, *C. mucosalis*, *C. showae, C. curvus*, *C. rectus*, *C. gracilis*, *C. ureolyticus*, *C. hyointestinalis*, *C. sputorum*, *C. upsaliensis*, *C. lari*, *C. fetus*, *C. jejuni*, *C. helvaticus*, and *C. coli*, respectively. M indicates a marker lane of 50 bp DNA Ladder (GeneRuler, Thermo Scientific). C indicates positive controls of the epsilon mix, N indicates negative control, and X indicates that the lane is blank.
